# Supplementary material for: An atlas of human proximal epididymis reveals cell-specific functions and distinct roles for CFTR
Source: Life Sci Alliance. 2020 Aug 27;3(11):e202000744. doi: 10.26508/lsa.202000744 (PMC7471510; doi:10.26508/lsa.202000744)
Supplement: Supplementary file 1 [file LSA-2020-00744_TableS1.docx]

| **Sample** | **AXH009** | **AXH012** | **AXH014** | **Number of Unique Molecular Identifier (UMI)** |
| --- | --- | --- | --- | --- |
| Cluster 0 | 912 | 260 | 1020 | 72145355 |
| Cluster 1 | 839 | 42 | 488 | 46863735 |
| Cluster 2 | 9 | 691 | 370 | 13834369 |
| Cluster 3 | 36 | 99 | 80 | 2923177 |
| Cluster 4 | 44 | 66 | 100 | 3310812 |
| Cluster 5 | 3 | 118 | 44 | 2143215 |
| Cluster 6 | 27 | 4 | 8 | 549118 |
| Cluster 7 | 6 | 29 | 4 | 422663 |
| Sum | 1876 | 1309 | 2114 | 142192444 |

**Supplementary Table 1.** Cell numbers in each cluster by donor with number of Unique Molecular Identifiers (UMI) captured in the combined clusters.
