# Supplementary material for: An atlas of human proximal epididymis reveals cell-specific functions and distinct roles for CFTR
Source: Life Sci Alliance. 2020 Aug 27;3(11):e202000744. doi: 10.26508/lsa.202000744 (PMC7471510; doi:10.26508/lsa.202000744)
Supplement: Supplementary file 2 [file LSA-2020-00744_TableS2.docx]

Supplementary Table 2. Marker gene list for each cluster ranked by power (cut off at 0.75).

| Index | gene | myAUC | avg_diff | power | pct.1 | pct.2 | cluster |
| --- | --- | --- | --- | --- | --- | --- | --- |
| 1 | SPAG11B | 0.991 | 2.056274 | 0.982 | 1 | 0.996 | 0 |
| 2 | CST11 | 0.987 | 2.41054 | 0.974 | 0.993 | 0.54 | 0 |
| 3 | SPAG11A | 0.986 | 1.771844 | 0.972 | 1 | 0.926 | 0 |
| 4 | LCN6 | 0.986 | 1.711215 | 0.972 | 1 | 0.916 | 0 |
| 5 | LCN8 | 0.983 | 1.248356 | 0.966 | 1 | 0.977 | 0 |
| 6 | DEFB119 | 0.982 | 3.103976 | 0.964 | 0.992 | 0.764 | 0 |
| 7 | DEFB118 | 0.981 | 1.760835 | 0.962 | 1 | 0.821 | 0 |
| 8 | LCN12 | 0.976 | 1.390187 | 0.952 | 1 | 0.965 | 0 |
| 9 | TEDDM1 | 0.963 | 1.269301 | 0.926 | 0.999 | 0.741 | 0 |
| 10 | PEBP4 | 0.96 | 1.335792 | 0.92 | 0.983 | 0.679 | 0 |
| 11 | DEFB121 | 0.959 | 2.709189 | 0.918 | 0.969 | 0.653 | 0 |
| 12 | CLPSL2 | 0.954 | 1.447067 | 0.908 | 0.999 | 0.903 | 0 |
| 13 | GPR64 | 0.948 | 0.882228 | 0.896 | 1 | 0.978 | 0 |
| 14 | YJEFN3 | 0.946 | 1.012936 | 0.892 | 0.956 | 0.659 | 0 |
| 15 | DEFB123 | 0.943 | 0.908629 | 0.886 | 0.919 | 0.412 | 0 |
| 16 | SERPINF2 | 0.941 | 0.923123 | 0.882 | 0.997 | 0.842 | 0 |
| 17 | TTC36 | 0.94 | 0.957868 | 0.88 | 0.992 | 0.756 | 0 |
| 18 | RNASE13 | 0.939 | 1.207836 | 0.878 | 0.978 | 0.64 | 0 |
| 19 | GGCT | 0.938 | 0.803555 | 0.876 | 0.996 | 0.876 | 0 |
| 20 | LARP6 | 0.926 | 0.775255 | 0.852 | 0.998 | 0.917 | 0 |
| 21 | CD320 | 0.925 | 0.812747 | 0.85 | 0.998 | 0.913 | 0 |
| 22 | NDRG2 | 0.916 | 0.771217 | 0.832 | 0.99 | 0.69 | 0 |
| 23 | FBXO2 | 0.915 | 0.688035 | 0.83 | 0.95 | 0.616 | 0 |
| 24 | UBXN11 | 0.912 | 0.796953 | 0.824 | 0.988 | 0.774 | 0 |
| 25 | LRCOL1 | 0.912 | 0.771432 | 0.824 | 1 | 0.882 | 0 |
| 26 | DEFB128 | 0.91 | 1.25421 | 0.82 | 0.983 | 0.731 | 0 |
| 27 | CRIP1 | 0.91 | 1.028869 | 0.82 | 0.998 | 0.795 | 0 |
| 28 | MYEOV2 | 0.909 | 0.61131 | 0.818 | 1 | 0.994 | 0 |
| 29 | SH3BGR | 0.908 | 0.70393 | 0.816 | 0.945 | 0.698 | 0 |
| 30 | PTGDS | 0.906 | 1.412598 | 0.812 | 1 | 0.958 | 0 |
| 31 | DBI | 0.906 | 0.745967 | 0.812 | 1 | 0.995 | 0 |
| 32 | ASS1 | 0.906 | 0.706885 | 0.812 | 0.971 | 0.664 | 0 |
| 33 | EPPIN | 0.902 | 1.024936 | 0.804 | 0.907 | 0.527 | 0 |
| 34 | SLC27A3 | 0.899 | 0.806442 | 0.798 | 0.999 | 0.767 | 0 |
| 35 | C19orf77 | 0.898 | 0.673365 | 0.796 | 1 | 0.967 | 0 |
| 36 | BLVRA | 0.896 | 0.978416 | 0.792 | 1 | 0.873 | 0 |
| 37 | GAMT | 0.895 | 0.586446 | 0.79 | 1 | 0.98 | 0 |
| 38 | C1orf122 | 0.892 | 0.633282 | 0.784 | 0.996 | 0.931 | 0 |
| 39 | MSMO1 | 0.89 | 0.90888 | 0.78 | 0.996 | 0.722 | 0 |
| 40 | AP1S2 | 0.89 | 0.883192 | 0.78 | 1 | 0.878 | 0 |
| 41 | HIST2H2BE | 0.89 | 0.788053 | 0.78 | 0.969 | 0.64 | 0 |
| 42 | RCN1 | 0.89 | 0.696574 | 0.78 | 1 | 0.936 | 0 |
| 43 | SCXA | 0.884 | 0.807205 | 0.768 | 0.94 | 0.64 | 0 |
| 44 | CST9L | 0.88 | 0.778094 | 0.76 | 0.932 | 0.708 | 0 |
| 45 | INSIG1 | 0.879 | 0.823558 | 0.758 | 0.987 | 0.765 | 0 |
| 46 | MTCH1 | 0.879 | 0.620274 | 0.758 | 1 | 0.977 | 0 |
| 47 | HOPX | 0.877 | 0.905077 | 0.754 | 1 | 0.855 | 0 |
| 48 | RBM11 | 0.877 | 0.67182 | 0.754 | 0.989 | 0.695 | 0 |

| 49 | NDUFA13 | 0.877 | 0.577185 | 0.754 | 1 | 0.996 | 0 |
| --- | --- | --- | --- | --- | --- | --- | --- |
| 50 | IDI1 | 0.876 | 0.925285 | 0.752 | 0.995 | 0.764 | 0 |
| 51 | EDDM3A | 0.993 | 2.347838 | 0.986 | 0.999 | 0.754 | 1 |
| 52 | EDDM3B | 0.992 | 3.198467 | 0.984 | 0.996 | 0.637 | 1 |
| 53 | WFDC8 | 0.99 | 1.705017 | 0.98 | 0.999 | 0.809 | 1 |
| 54 | CRISP1 | 0.987 | 2.802778 | 0.974 | 0.991 | 0.583 | 1 |
| 55 | WFDC9 | 0.986 | 1.731281 | 0.972 | 1 | 0.851 | 1 |
| 56 | CLPSL1 | 0.985 | 1.795873 | 0.97 | 1 | 0.823 | 1 |
| 57 | PAX2 | 0.984 | 1.285723 | 0.968 | 1 | 0.721 | 1 |
| 58 | DEFB132 | 0.981 | 1.714338 | 0.962 | 0.999 | 0.805 | 1 |
| 59 | DEFB129 | 0.969 | 2.107967 | 0.938 | 0.988 | 0.707 | 1 |
| 60 | ITM2B | 0.969 | 1.020015 | 0.938 | 1 | 1 | 1 |
| 61 | GSTM3 | 0.967 | 1.345564 | 0.934 | 1 | 0.863 | 1 |
| 62 | PDE6A | 0.959 | 0.942619 | 0.918 | 0.969 | 0.59 | 1 |
| 63 | RP11-793H | 0.958 | 1.50142 | 0.916 | 0.999 | 0.835 | 1 |
| 64 | NPFF | 0.958 | 1.49254 | 0.916 | 1 | 0.84 | 1 |
| 65 | SCGB1D2 | 0.956 | 1.214931 | 0.912 | 0.996 | 0.773 | 1 |
| 66 | CAMP | 0.955 | 1.897382 | 0.91 | 0.995 | 0.773 | 1 |
| 67 | CD52 | 0.952 | 1.218835 | 0.904 | 1 | 0.997 | 1 |
| 68 | TFAP2B | 0.95 | 0.804772 | 0.9 | 0.985 | 0.646 | 1 |
| 69 | AP1S3 | 0.95 | 0.738302 | 0.9 | 0.955 | 0.415 | 1 |
| 70 | SPINK13 | 0.946 | 1.377291 | 0.892 | 0.991 | 0.612 | 1 |
| 71 | PCP4 | 0.944 | 1.054863 | 0.888 | 1 | 0.926 | 1 |
| 72 | TMEM150 | 0.942 | 0.855466 | 0.884 | 0.988 | 0.748 | 1 |
| 73 | CAMK2B | 0.941 | 0.947363 | 0.882 | 0.98 | 0.628 | 1 |
| 74 | PCCA | 0.933 | 1.270494 | 0.866 | 0.999 | 0.868 | 1 |
| 75 | REG3G | 0.932 | 0.789349 | 0.864 | 0.923 | 0.42 | 1 |
| 76 | DAPL1 | 0.929 | 1.209033 | 0.858 | 0.983 | 0.67 | 1 |
| 77 | ELSPBP1 | 0.928 | 1.120897 | 0.856 | 1 | 0.905 | 1 |
| 78 | RP11-527L | 0.928 | 1.007019 | 0.856 | 1 | 0.874 | 1 |
| 79 | CAPN3 | 0.926 | 0.954281 | 0.852 | 0.927 | 0.412 | 1 |
| 80 | SPINT4 | 0.925 | 1.488659 | 0.85 | 0.92 | 0.426 | 1 |
| 81 | RP11-304L | 0.923 | 1.040474 | 0.846 | 1 | 0.962 | 1 |
| 82 | EME1 | 0.921 | 0.723132 | 0.842 | 0.943 | 0.607 | 1 |
| 83 | MIR205HG | 0.918 | 0.724244 | 0.836 | 0.999 | 0.779 | 1 |
| 84 | AC011298. | 0.914 | 0.673353 | 0.828 | 0.901 | 0.528 | 1 |
| 85 | WFDC2 | 0.913 | 1.10738 | 0.826 | 1 | 0.987 | 1 |
| 86 | MAPK12 | 0.912 | 0.517426 | 0.824 | 0.906 | 0.507 | 1 |
| 87 | RP11-301G | 0.908 | 0.462097 | 0.816 | 0.904 | 0.465 | 1 |
| 88 | DEFB127 | 0.907 | 1.120781 | 0.814 | 0.994 | 0.753 | 1 |
| 89 | PATE2 | 0.905 | 0.881209 | 0.81 | 1 | 0.823 | 1 |
| 90 | IL1R2 | 0.905 | 0.836738 | 0.81 | 0.979 | 0.638 | 1 |
| 91 | APOC1 | 0.905 | 0.796264 | 0.81 | 0.919 | 0.496 | 1 |
| 92 | BMX | 0.905 | 0.704265 | 0.81 | 0.993 | 0.764 | 1 |
| 93 | RP11-635N | 0.904 | 0.683645 | 0.808 | 0.977 | 0.759 | 1 |
| 94 | TEX30 | 0.901 | 0.673118 | 0.802 | 0.989 | 0.732 | 1 |
| 95 | HMGN5 | 0.9 | 0.802931 | 0.8 | 0.994 | 0.894 | 1 |
| 96 | MARCKSL1 | 0.899 | 0.712213 | 0.798 | 1 | 0.958 | 1 |
| 97 | DCXR | 0.898 | 0.724419 | 0.796 | 1 | 0.992 | 1 |
| 98 | FCGR1A | 0.895 | 0.555219 | 0.79 | 0.947 | 0.672 | 1 |

| 99 | LINC00844 | 0.893 | 0.835758 | 0.786 | 0.977 | 0.768 | 1 |
| --- | --- | --- | --- | --- | --- | --- | --- |
| 100 | ID3 | 0.891 | 0.955407 | 0.782 | 0.996 | 0.823 | 1 |
| 101 | RP13-650J | 0.889 | 0.567963 | 0.778 | 0.937 | 0.697 | 1 |
| 102 | RP11-326C | 0.889 | 0.55825 | 0.778 | 0.97 | 0.744 | 1 |
| 103 | CHN1 | 0.888 | 0.51537 | 0.776 | 0.888 | 0.435 | 1 |
| 104 | FKBP2 | 0.883 | 0.545076 | 0.766 | 1 | 0.996 | 1 |
| 105 | RP3-395M | 0.883 | 0.498544 | 0.766 | 0.903 | 0.491 | 1 |
| 106 | XX-C2158C | 0.881 | 0.554537 | 0.762 | 0.869 | 0.441 | 1 |
| 107 | ALB | 0.88 | 0.451235 | 0.76 | 0.874 | 0.381 | 1 |
| 108 | PATE3 | 0.879 | 0.661011 | 0.758 | 0.904 | 0.596 | 1 |
| 109 | DAXX | 0.877 | 0.696733 | 0.754 | 0.953 | 0.65 | 1 |
| 110 | ABRACL | 0.876 | 0.614571 | 0.752 | 0.995 | 0.907 | 1 |
| 111 | SGSM2 | 0.875 | 0.557381 | 0.75 | 0.909 | 0.542 | 1 |
| 112 | SERPINA1 | 0.98 | 2.685458 | 0.96 | 0.999 | 0.766 | 2 |
| 113 | ASRGL1 | 0.975 | 1.679 | 0.95 | 0.998 | 0.601 | 2 |
| 114 | TMEM176 | 0.975 | 1.502958 | 0.95 | 0.991 | 0.54 | 2 |
| 115 | AMN | 0.971 | 1.427776 | 0.942 | 0.987 | 0.334 | 2 |
| 116 | TMEM176 | 0.971 | 1.410281 | 0.942 | 0.992 | 0.604 | 2 |
| 117 | TGM2 | 0.965 | 1.707142 | 0.93 | 0.995 | 0.697 | 2 |
| 118 | CTSH | 0.965 | 1.615489 | 0.93 | 0.992 | 0.761 | 2 |
| 119 | FABP3 | 0.962 | 1.352108 | 0.924 | 0.983 | 0.601 | 2 |
| 120 | LGALS2 | 0.959 | 1.270039 | 0.918 | 0.974 | 0.598 | 2 |
| 121 | GCHFR | 0.959 | 1.166909 | 0.918 | 0.991 | 0.628 | 2 |
| 122 | DMKN | 0.956 | 1.50522 | 0.912 | 0.999 | 0.842 | 2 |
| 123 | DPEP1 | 0.956 | 0.958907 | 0.912 | 0.964 | 0.487 | 2 |
| 124 | ADIRF | 0.955 | 2.108571 | 0.91 | 0.999 | 0.723 | 2 |
| 125 | CA12 | 0.953 | 1.10548 | 0.906 | 0.982 | 0.619 | 2 |
| 126 | DEFB1 | 0.951 | 1.053935 | 0.902 | 0.973 | 0.434 | 2 |
| 127 | FXYD2 | 0.948 | 1.734716 | 0.896 | 0.963 | 0.527 | 2 |
| 128 | CCDC146 | 0.947 | 1.294621 | 0.894 | 0.993 | 0.667 | 2 |
| 129 | FYN | 0.947 | 1.148293 | 0.894 | 0.98 | 0.615 | 2 |
| 130 | SLC51B | 0.945 | 1.107648 | 0.89 | 0.961 | 0.569 | 2 |
| 131 | RBP5 | 0.944 | 0.976434 | 0.888 | 0.963 | 0.423 | 2 |
| 132 | DCDC2 | 0.943 | 1.091104 | 0.886 | 0.993 | 0.689 | 2 |
| 133 | PRAP1 | 0.939 | 1.099891 | 0.878 | 0.939 | 0.573 | 2 |
| 134 | FTL | 0.936 | 1.332142 | 0.872 | 1 | 1 | 2 |
| 135 | AZGP1 | 0.935 | 0.930019 | 0.87 | 0.943 | 0.522 | 2 |
| 136 | APOE | 0.933 | 1.663741 | 0.866 | 0.977 | 0.637 | 2 |
| 137 | AGT | 0.932 | 0.854823 | 0.864 | 0.948 | 0.518 | 2 |
| 138 | NAPSA | 0.929 | 1.225075 | 0.858 | 0.95 | 0.534 | 2 |
| 139 | CDKN2C | 0.925 | 0.76256 | 0.85 | 0.938 | 0.535 | 2 |
| 140 | SLC39A5 | 0.923 | 0.687885 | 0.846 | 0.931 | 0.571 | 2 |
| 141 | IGFBP4 | 0.92 | 1.138883 | 0.84 | 0.987 | 0.598 | 2 |
| 142 | ESR1 | 0.92 | 1.009745 | 0.84 | 0.935 | 0.487 | 2 |
| 143 | CST3 | 0.916 | 1.380995 | 0.832 | 1 | 1 | 2 |
| 144 | ZCCHC12 | 0.912 | 0.797002 | 0.824 | 0.92 | 0.573 | 2 |
| 145 | RP11-59E1 | 0.912 | 0.702712 | 0.824 | 0.903 | 0.448 | 2 |
| 146 | MGLL | 0.91 | 0.729155 | 0.82 | 0.947 | 0.406 | 2 |
| 147 | TIMP3 | 0.909 | 1.221863 | 0.818 | 0.991 | 0.76 | 2 |
| 148 | RP11-320N | 0.909 | 0.624479 | 0.818 | 0.916 | 0.521 | 2 |

| 149 | CXCL14 | 0.908 | 1.203911 | 0.816 | 0.981 | 0.685 | 2 |
| --- | --- | --- | --- | --- | --- | --- | --- |
| 150 | GNAO1 | 0.908 | 0.862109 | 0.816 | 0.916 | 0.442 | 2 |
| 151 | HPN | 0.908 | 0.823286 | 0.816 | 0.952 | 0.589 | 2 |
| 152 | TPM2 | 0.908 | 0.586748 | 0.816 | 0.917 | 0.414 | 2 |
| 153 | GNG11 | 0.907 | 1.155101 | 0.814 | 0.963 | 0.601 | 2 |
| 154 | PGR | 0.907 | 0.793043 | 0.814 | 0.907 | 0.51 | 2 |
| 155 | SLC47A1 | 0.907 | 0.762293 | 0.814 | 0.915 | 0.58 | 2 |
| 156 | CUBN | 0.907 | 0.670133 | 0.814 | 0.901 | 0.394 | 2 |
| 157 | VDR | 0.907 | 0.613432 | 0.814 | 0.911 | 0.485 | 2 |
| 158 | NSG1 | 0.906 | 0.910861 | 0.812 | 0.927 | 0.525 | 2 |
| 159 | DEGS2 | 0.906 | 0.637986 | 0.812 | 0.901 | 0.433 | 2 |
| 160 | PCK2 | 0.905 | 0.724668 | 0.81 | 0.903 | 0.41 | 2 |
| 161 | PDK4 | 0.904 | 1.36682 | 0.808 | 0.952 | 0.454 | 2 |
| 162 | SULT1A2 | 0.903 | 0.512813 | 0.806 | 0.862 | 0.329 | 2 |
| 163 | RAB31 | 0.902 | 0.774553 | 0.804 | 0.931 | 0.435 | 2 |
| 164 | GPX3 | 0.901 | 1.083847 | 0.802 | 0.934 | 0.519 | 2 |
| 165 | HSD17B14 | 0.9 | 0.716188 | 0.8 | 0.935 | 0.499 | 2 |
| 166 | PDZK1IP1 | 0.897 | 1.16881 | 0.794 | 0.905 | 0.434 | 2 |
| 167 | SLC9A3R1 | 0.897 | 0.812316 | 0.794 | 0.959 | 0.646 | 2 |
| 168 | FBLN1 | 0.895 | 0.744526 | 0.79 | 0.943 | 0.507 | 2 |
| 169 | ANPEP | 0.894 | 0.529442 | 0.788 | 0.882 | 0.47 | 2 |
| 170 | CLDN2 | 0.892 | 0.990165 | 0.784 | 0.969 | 0.793 | 2 |
| 171 | LGALS4 | 0.892 | 0.632493 | 0.784 | 0.88 | 0.553 | 2 |
| 172 | CFI | 0.891 | 0.909325 | 0.782 | 0.958 | 0.657 | 2 |
| 173 | EPHX2 | 0.89 | 0.69483 | 0.78 | 0.932 | 0.503 | 2 |
| 174 | PTMS | 0.889 | 0.757663 | 0.778 | 0.977 | 0.546 | 2 |
| 175 | RGN | 0.888 | 0.699205 | 0.776 | 0.903 | 0.539 | 2 |
| 176 | FCGRT | 0.887 | 0.827004 | 0.774 | 0.979 | 0.729 | 2 |
| 177 | OAF | 0.887 | 0.58859 | 0.774 | 0.918 | 0.399 | 2 |
| 178 | S100A14 | 0.886 | 0.953889 | 0.772 | 0.921 | 0.412 | 2 |
| 179 | MT1F | 0.883 | 1.188951 | 0.766 | 0.907 | 0.51 | 2 |
| 180 | SERPINE2 | 0.881 | 0.926878 | 0.762 | 0.893 | 0.486 | 2 |
| 181 | CFTR | 0.878 | 0.347475 | 0.756 | 0.81 | 0.251 | 2 |
| 182 | ALDH2 | 0.877 | 0.77719 | 0.754 | 0.922 | 0.586 | 2 |
| 183 | DDAH2 | 0.877 | 0.672327 | 0.754 | 0.94 | 0.538 | 2 |
| 184 | SLC44A4 | 0.877 | 0.596894 | 0.754 | 0.893 | 0.497 | 2 |
| 185 | CCDC170 | 0.876 | 0.419416 | 0.752 | 0.917 | 0.614 | 2 |
| 186 | PPP1R1A | 0.876 | 0.360557 | 0.752 | 0.835 | 0.339 | 2 |
| 187 | KRT5 | 0.998 | 2.056539 | 0.996 | 1 | 0.638 | 3 |
| 188 | CSTA | 0.998 | 1.523889 | 0.996 | 1 | 0.412 | 3 |
| 189 | TACSTD2 | 0.997 | 2.595191 | 0.994 | 1 | 0.573 | 3 |
| 190 | DST | 0.996 | 2.517907 | 0.992 | 1 | 0.566 | 3 |
| 191 | MIR205HG | 0.994 | 2.064555 | 0.988 | 1 | 0.829 | 3 |
| 192 | KRT17 | 0.99 | 1.736267 | 0.98 | 0.991 | 0.257 | 3 |
| 193 | CXCL14 | 0.986 | 2.474388 | 0.972 | 1 | 0.734 | 3 |
| 194 | FXYD3 | 0.986 | 1.15458 | 0.972 | 0.981 | 0.31 | 3 |
| 195 | CLDN1 | 0.985 | 1.650825 | 0.97 | 0.995 | 0.586 | 3 |
| 196 | ANXA1 | 0.982 | 2.180266 | 0.964 | 1 | 0.329 | 3 |
| 197 | HSPA5 | 0.981 | 1.524028 | 0.962 | 1 | 0.995 | 3 |
| 198 | ZFP36L2 | 0.98 | 1.733119 | 0.96 | 1 | 0.768 | 3 |

| 199 | LIMA1 | 0.98 | 1.571627 | 0.96 | 0.995 | 0.788 | 3 |
| --- | --- | --- | --- | --- | --- | --- | --- |
| 200 | FN1 | 0.98 | 1.182221 | 0.96 | 0.995 | 0.565 | 3 |
| 201 | GADD45A | 0.973 | 1.597758 | 0.946 | 1 | 0.921 | 3 |
| 202 | CEBPD | 0.972 | 1.771167 | 0.944 | 1 | 0.903 | 3 |
| 203 | ITGA2 | 0.969 | 1.272171 | 0.938 | 0.977 | 0.501 | 3 |
| 204 | PLAU | 0.966 | 1.640553 | 0.932 | 0.967 | 0.198 | 3 |
| 205 | ZFP36 | 0.966 | 1.523399 | 0.932 | 1 | 0.956 | 3 |
| 206 | TNFAIP3 | 0.965 | 1.653756 | 0.93 | 0.991 | 0.479 | 3 |
| 207 | TP63 | 0.965 | 0.845166 | 0.93 | 0.944 | 0.35 | 3 |
| 208 | CYR61 | 0.964 | 1.844362 | 0.928 | 1 | 0.907 | 3 |
| 209 | RAP2B | 0.958 | 1.250709 | 0.916 | 0.967 | 0.324 | 3 |
| 210 | SOCS3 | 0.957 | 1.358916 | 0.914 | 0.986 | 0.319 | 3 |
| 211 | FOSB | 0.956 | 1.467547 | 0.912 | 1 | 0.941 | 3 |
| 212 | PKP1 | 0.953 | 0.675712 | 0.906 | 0.926 | 0.356 | 3 |
| 213 | IER3 | 0.952 | 1.733976 | 0.904 | 1 | 0.665 | 3 |
| 214 | NFKBIA | 0.952 | 1.494447 | 0.904 | 1 | 0.938 | 3 |
| 215 | FLNA | 0.95 | 0.917299 | 0.9 | 0.972 | 0.437 | 3 |
| 216 | KLF5 | 0.948 | 1.269328 | 0.896 | 0.967 | 0.563 | 3 |
| 217 | COMP | 0.948 | 0.954244 | 0.896 | 0.935 | 0.208 | 3 |
| 218 | S100A6 | 0.947 | 1.56498 | 0.894 | 0.995 | 0.576 | 3 |
| 219 | IL8 | 0.945 | 2.141985 | 0.89 | 0.981 | 0.541 | 3 |
| 220 | IGFBP6 | 0.942 | 1.030531 | 0.884 | 0.94 | 0.274 | 3 |
| 221 | MEG3 | 0.941 | 1.221943 | 0.882 | 0.921 | 0.306 | 3 |
| 222 | SOD2 | 0.936 | 1.516588 | 0.872 | 0.995 | 0.779 | 3 |
| 223 | H1FX | 0.935 | 1.142765 | 0.87 | 0.995 | 0.87 | 3 |
| 224 | IGFBP2 | 0.933 | 1.012537 | 0.866 | 0.995 | 0.652 | 3 |
| 225 | SNCG | 0.93 | 1.107416 | 0.86 | 1 | 0.504 | 3 |
| 226 | TRIM29 | 0.929 | 0.623034 | 0.858 | 0.893 | 0.327 | 3 |
| 227 | S100A2 | 0.927 | 2.169771 | 0.854 | 0.916 | 0.202 | 3 |
| 228 | SERPINF1 | 0.927 | 0.780323 | 0.854 | 0.935 | 0.387 | 3 |
| 229 | GDF15 | 0.923 | 1.806661 | 0.846 | 0.949 | 0.59 | 3 |
| 230 | SFRP5 | 0.923 | 0.622601 | 0.846 | 0.888 | 0.446 | 3 |
| 231 | FILIP1L | 0.922 | 0.846496 | 0.844 | 0.926 | 0.474 | 3 |
| 232 | COL7A1 | 0.921 | 0.646724 | 0.842 | 0.898 | 0.357 | 3 |
| 233 | C16orf74 | 0.921 | 0.52829 | 0.842 | 0.879 | 0.346 | 3 |
| 234 | SYNPO2 | 0.92 | 0.580259 | 0.84 | 0.879 | 0.341 | 3 |
| 235 | TNS4 | 0.919 | 0.817952 | 0.838 | 0.893 | 0.373 | 3 |
| 236 | GBP1 | 0.918 | 1.358784 | 0.836 | 0.953 | 0.493 | 3 |
| 237 | AMIGO2 | 0.917 | 0.717864 | 0.834 | 0.93 | 0.457 | 3 |
| 238 | CDKN1A | 0.916 | 0.914036 | 0.832 | 0.995 | 0.919 | 3 |
| 239 | NDRG4 | 0.916 | 0.589872 | 0.832 | 0.874 | 0.37 | 3 |
| 240 | NCOA7 | 0.915 | 1.173781 | 0.83 | 0.991 | 0.612 | 3 |
| 241 | ETS2 | 0.915 | 0.996772 | 0.83 | 0.967 | 0.657 | 3 |
| 242 | TPM4 | 0.915 | 0.963276 | 0.83 | 0.949 | 0.634 | 3 |
| 243 | NUAK1 | 0.914 | 0.459627 | 0.828 | 0.879 | 0.307 | 3 |
| 244 | ZFP36L1 | 0.912 | 1.099833 | 0.824 | 1 | 0.996 | 3 |
| 245 | CRABP2 | 0.911 | 1.072309 | 0.822 | 0.902 | 0.342 | 3 |
| 246 | SOD3 | 0.907 | 0.895123 | 0.814 | 1 | 0.623 | 3 |
| 247 | SYT8 | 0.907 | 0.696723 | 0.814 | 0.847 | 0.357 | 3 |
| 248 | TFPI2 | 0.906 | 1.654682 | 0.812 | 0.921 | 0.275 | 3 |

| 249 | IRF1 | 0.905 | 1.090077 | 0.81 | 0.981 | 0.68 | 3 |
| --- | --- | --- | --- | --- | --- | --- | --- |
| 250 | TIMP3 | 0.904 | 1.262198 | 0.808 | 1 | 0.798 | 3 |
| 251 | BTG1 | 0.904 | 0.925322 | 0.808 | 1 | 0.976 | 3 |
| 252 | CALML3 | 0.904 | 0.501851 | 0.808 | 0.856 | 0.348 | 3 |
| 253 | PLTP | 0.903 | 0.714362 | 0.806 | 0.94 | 0.468 | 3 |
| 254 | MT1X | 0.902 | 1.287405 | 0.804 | 0.995 | 0.94 | 3 |
| 255 | CNN2 | 0.902 | 0.746865 | 0.804 | 0.944 | 0.414 | 3 |
| 256 | ARL4C | 0.9 | 0.73147 | 0.8 | 0.958 | 0.586 | 3 |
| 257 | RND3 | 0.899 | 0.893323 | 0.798 | 0.926 | 0.358 | 3 |
| 258 | USP31 | 0.898 | 0.565865 | 0.796 | 0.86 | 0.326 | 3 |
| 259 | AQP3 | 0.898 | 0.469782 | 0.796 | 0.828 | 0.275 | 3 |
| 260 | DHRS3 | 0.897 | 0.742402 | 0.794 | 0.93 | 0.405 | 3 |
| 261 | SPINK5 | 0.897 | 0.492118 | 0.794 | 0.819 | 0.14 | 3 |
| 262 | GADD45B | 0.896 | 1.214611 | 0.792 | 1 | 0.948 | 3 |
| 263 | SGCA | 0.896 | 0.257363 | 0.792 | 0.763 | 0.125 | 3 |
| 264 | PMAIP1 | 0.895 | 0.869239 | 0.79 | 0.953 | 0.57 | 3 |
| 265 | MATN2 | 0.895 | 0.624387 | 0.79 | 0.86 | 0.349 | 3 |
| 266 | KRT19 | 0.894 | 1.20888 | 0.788 | 0.981 | 0.849 | 3 |
| 267 | LAMB3 | 0.894 | 0.927222 | 0.788 | 0.912 | 0.525 | 3 |
| 268 | NR4A1 | 0.893 | 1.051116 | 0.786 | 1 | 0.954 | 3 |
| 269 | PCDH9 | 0.893 | 0.516475 | 0.786 | 0.87 | 0.526 | 3 |
| 270 | FGFR2 | 0.891 | 0.531166 | 0.782 | 0.865 | 0.384 | 3 |
| 271 | BBC3 | 0.888 | 0.922996 | 0.776 | 0.898 | 0.421 | 3 |
| 272 | JAG1 | 0.887 | 0.513674 | 0.774 | 0.884 | 0.369 | 3 |
| 273 | EGR3 | 0.886 | 0.732519 | 0.772 | 0.921 | 0.579 | 3 |
| 274 | ERRFI1 | 0.885 | 0.945034 | 0.77 | 0.926 | 0.649 | 3 |
| 275 | LMO4 | 0.883 | 0.758746 | 0.766 | 0.944 | 0.598 | 3 |
| 276 | JUNB | 0.882 | 1.009881 | 0.764 | 1 | 0.992 | 3 |
| 277 | FRMD4B | 0.881 | 0.978572 | 0.762 | 0.935 | 0.518 | 3 |
| 278 | BCL3 | 0.881 | 0.498148 | 0.762 | 0.902 | 0.308 | 3 |
| 279 | FABP5 | 0.881 | 0.484773 | 0.762 | 0.833 | 0.225 | 3 |
| 280 | NUPR1 | 0.878 | 0.941545 | 0.756 | 1 | 0.886 | 3 |
| 281 | BAZ1A | 0.877 | 0.766726 | 0.754 | 0.944 | 0.715 | 3 |
| 282 | LMO2 | 0.877 | 0.432269 | 0.754 | 0.856 | 0.273 | 3 |
| 283 | PHYHIPL | 0.877 | 0.329855 | 0.754 | 0.8 | 0.287 | 3 |
| 284 | EGR4 | 0.876 | 0.255846 | 0.752 | 0.726 | 0.158 | 3 |
| 285 | SPARCL1 | 0.996 | 2.949971 | 0.992 | 1 | 0.455 | 4 |
| 286 | IGFBP7 | 0.988 | 2.879334 | 0.976 | 0.995 | 0.737 | 4 |
| 287 | LGALS1 | 0.979 | 1.948973 | 0.958 | 0.995 | 0.442 | 4 |
| 288 | VIM | 0.965 | 2.379885 | 0.93 | 1 | 0.737 | 4 |
| 289 | BST2 | 0.96 | 1.506405 | 0.92 | 0.981 | 0.699 | 4 |
| 290 | S100A6 | 0.955 | 1.998689 | 0.91 | 0.995 | 0.577 | 4 |
| 291 | TMSB4X | 0.955 | 1.269911 | 0.91 | 1 | 0.999 | 4 |
| 292 | C11orf96 | 0.953 | 2.26843 | 0.906 | 0.938 | 0.261 | 4 |
| 293 | GSN | 0.95 | 1.515274 | 0.9 | 0.952 | 0.433 | 4 |
| 294 | PTMS | 0.938 | 1.196133 | 0.876 | 0.995 | 0.618 | 4 |
| 295 | VAMP5 | 0.935 | 1.244445 | 0.87 | 0.943 | 0.474 | 4 |
| 296 | KLF2 | 0.928 | 1.651932 | 0.856 | 0.914 | 0.378 | 4 |
| 297 | TAGLN | 0.927 | 1.969337 | 0.854 | 0.886 | 0.295 | 4 |
| 298 | MYL9 | 0.925 | 1.594106 | 0.85 | 0.962 | 0.436 | 4 |

| 299 | SELM | 0.925 | 1.166435 | 0.85 | 0.957 | 0.54 | 4 |
| --- | --- | --- | --- | --- | --- | --- | --- |
| 300 | TM4SF1 | 0.918 | 1.950015 | 0.836 | 0.838 | 0.185 | 4 |
| 301 | MGP | 0.915 | 1.65729 | 0.83 | 0.952 | 0.559 | 4 |
| 302 | GNG11 | 0.914 | 1.353303 | 0.828 | 0.981 | 0.661 | 4 |
| 303 | PRKCDBP | 0.913 | 0.726179 | 0.826 | 0.881 | 0.4 | 4 |
| 304 | CAV1 | 0.907 | 1.362357 | 0.814 | 0.9 | 0.344 | 4 |
| 305 | SPARC | 0.906 | 1.234199 | 0.812 | 0.876 | 0.387 | 4 |
| 306 | A2M | 0.896 | 1.507468 | 0.792 | 0.843 | 0.337 | 4 |
| 307 | JUNB | 0.895 | 1.044804 | 0.79 | 1 | 0.992 | 4 |
| 308 | LY6E | 0.892 | 0.962179 | 0.784 | 0.995 | 0.692 | 4 |
| 309 | PTRF | 0.889 | 0.910349 | 0.778 | 0.871 | 0.381 | 4 |
| 310 | TAGLN2 | 0.888 | 1.179358 | 0.776 | 0.929 | 0.623 | 4 |
| 311 | NFKBIA | 0.884 | 1.370593 | 0.768 | 0.995 | 0.938 | 4 |
| 312 | IGFBP4 | 0.883 | 1.053363 | 0.766 | 0.971 | 0.664 | 4 |
| 313 | SOCS3 | 0.882 | 1.712544 | 0.764 | 0.876 | 0.325 | 4 |
| 314 | PLAC9 | 0.881 | 0.67978 | 0.762 | 0.79 | 0.294 | 4 |
| 315 | ZFP36 | 0.88 | 1.170687 | 0.76 | 1 | 0.956 | 4 |
| 316 | ADIRF | 0.877 | 1.271474 | 0.754 | 0.99 | 0.77 | 4 |
| 317 | COX7A1 | 0.876 | 0.890221 | 0.752 | 0.848 | 0.296 | 4 |
| 318 | IGF2 | 0.876 | 0.53464 | 0.752 | 0.71 | 0.17 | 4 |
| 319 | CAPS | 1 | 3.071222 | 1 | 1 | 0.397 | 5 |
| 320 | AK1 | 0.999 | 2.728222 | 0.998 | 1 | 0.703 | 5 |
| 321 | C1orf194 | 0.999 | 2.491117 | 0.998 | 1 | 0.358 | 5 |
| 322 | C1orf192 | 0.999 | 1.675688 | 0.998 | 1 | 0.296 | 5 |
| 323 | ODF3B | 0.997 | 1.638772 | 0.994 | 1 | 0.421 | 5 |
| 324 | C11orf88 | 0.997 | 1.442676 | 0.994 | 1 | 0.276 | 5 |
| 325 | FAM183A | 0.994 | 2.162446 | 0.988 | 0.994 | 0.382 | 5 |
| 326 | TPPP3 | 0.993 | 2.511938 | 0.986 | 0.994 | 0.419 | 5 |
| 327 | MNS1 | 0.993 | 1.593932 | 0.986 | 1 | 0.486 | 5 |
| 328 | LINC01171 | 0.992 | 1.322159 | 0.984 | 0.994 | 0.341 | 5 |
| 329 | LRRIQ1 | 0.99 | 1.48174 | 0.98 | 0.994 | 0.714 | 5 |
| 330 | C9orf24 | 0.989 | 2.56685 | 0.978 | 0.988 | 0.252 | 5 |
| 331 | FAM81B | 0.989 | 1.431396 | 0.978 | 0.994 | 0.441 | 5 |
| 332 | PIFO | 0.986 | 1.674231 | 0.972 | 0.994 | 0.582 | 5 |
| 333 | CCDC170 | 0.986 | 1.60242 | 0.972 | 1 | 0.664 | 5 |
| 334 | CCDC11 | 0.986 | 1.415521 | 0.972 | 1 | 0.638 | 5 |
| 335 | CCDC173 | 0.985 | 1.210796 | 0.97 | 0.988 | 0.55 | 5 |
| 336 | C9orf116 | 0.984 | 1.5313 | 0.968 | 0.994 | 0.722 | 5 |
| 337 | RSPH1 | 0.982 | 1.606363 | 0.964 | 0.988 | 0.634 | 5 |
| 338 | C20orf85 | 0.981 | 2.111635 | 0.962 | 0.982 | 0.373 | 5 |
| 339 | RP11-356K | 0.981 | 1.690343 | 0.962 | 0.976 | 0.359 | 5 |
| 340 | DYNLRB2 | 0.98 | 1.564155 | 0.96 | 0.994 | 0.75 | 5 |
| 341 | NWD1 | 0.98 | 1.029739 | 0.96 | 0.976 | 0.299 | 5 |
| 342 | C5orf49 | 0.978 | 1.42506 | 0.956 | 0.982 | 0.447 | 5 |
| 343 | C4orf48 | 0.977 | 1.416868 | 0.954 | 1 | 0.688 | 5 |
| 344 | GSTA3 | 0.976 | 0.856323 | 0.952 | 0.952 | 0.148 | 5 |
| 345 | MORN5 | 0.975 | 1.291093 | 0.95 | 0.982 | 0.448 | 5 |
| 346 | CKB | 0.973 | 1.736157 | 0.946 | 1 | 0.529 | 5 |
| 347 | KIAA1377 | 0.97 | 1.12969 | 0.94 | 0.994 | 0.407 | 5 |
| 348 | WDR96 | 0.968 | 1.145126 | 0.936 | 0.964 | 0.447 | 5 |

| 349 | ENKUR | 0.967 | 0.913147 | 0.934 | 0.964 | 0.481 | 5 |
| --- | --- | --- | --- | --- | --- | --- | --- |
| 350 | CCDC42B | 0.966 | 1.053804 | 0.932 | 0.939 | 0.301 | 5 |
| 351 | METRN | 0.965 | 1.328551 | 0.93 | 1 | 0.754 | 5 |
| 352 | FOXJ1 | 0.965 | 1.195578 | 0.93 | 1 | 0.497 | 5 |
| 353 | AKAP14 | 0.965 | 0.900071 | 0.93 | 0.933 | 0.092 | 5 |
| 354 | LRRC46 | 0.96 | 0.86045 | 0.92 | 0.958 | 0.424 | 5 |
| 355 | CCDC146 | 0.959 | 1.63348 | 0.918 | 1 | 0.724 | 5 |
| 356 | MT-CO2 | 0.956 | 1.281603 | 0.912 | 1 | 1 | 5 |
| 357 | CATSPERD | 0.955 | 0.843994 | 0.91 | 0.945 | 0.435 | 5 |
| 358 | IGFBP7 | 0.953 | 1.42004 | 0.906 | 1 | 0.739 | 5 |
| 359 | CCDC113 | 0.953 | 0.718858 | 0.906 | 0.939 | 0.365 | 5 |
| 360 | SPAG17 | 0.952 | 0.973732 | 0.904 | 0.964 | 0.494 | 5 |
| 361 | RIBC2 | 0.951 | 0.795329 | 0.902 | 0.921 | 0.36 | 5 |
| 362 | CCDC39 | 0.95 | 0.992472 | 0.9 | 0.939 | 0.215 | 5 |
| 363 | CAPSL | 0.949 | 1.298372 | 0.898 | 0.958 | 0.581 | 5 |
| 364 | DRC1 | 0.948 | 0.698184 | 0.896 | 0.897 | 0.125 | 5 |
| 365 | AC013264. | 0.947 | 0.788812 | 0.894 | 0.915 | 0.136 | 5 |
| 366 | SNTN | 0.943 | 1.336217 | 0.886 | 0.939 | 0.367 | 5 |
| 367 | CCDC181 | 0.942 | 0.804807 | 0.884 | 0.952 | 0.427 | 5 |
| 368 | C17orf72 | 0.942 | 0.752041 | 0.884 | 0.921 | 0.332 | 5 |
| 369 | DTHD1 | 0.941 | 0.582575 | 0.882 | 0.885 | 0.088 | 5 |
| 370 | NNAT | 0.939 | 1.502362 | 0.878 | 0.915 | 0.333 | 5 |
| 371 | CTGF | 0.938 | 1.470676 | 0.876 | 0.958 | 0.618 | 5 |
| 372 | SMIM5 | 0.937 | 0.527091 | 0.874 | 0.921 | 0.306 | 5 |
| 373 | RIIAD1 | 0.936 | 0.451389 | 0.872 | 0.897 | 0.198 | 5 |
| 374 | DNAH12 | 0.935 | 0.606096 | 0.87 | 0.891 | 0.2 | 5 |
| 375 | C1orf87 | 0.933 | 0.396503 | 0.866 | 0.855 | 0.035 | 5 |
| 376 | RSPH4A | 0.932 | 0.711602 | 0.864 | 0.891 | 0.317 | 5 |
| 377 | THY1 | 0.931 | 1.076622 | 0.862 | 0.921 | 0.407 | 5 |
| 378 | CSRP2 | 0.928 | 0.982289 | 0.856 | 0.964 | 0.635 | 5 |
| 379 | ATG9B | 0.927 | 0.58911 | 0.854 | 0.891 | 0.145 | 5 |
| 380 | IFI27 | 0.926 | 1.477381 | 0.852 | 0.939 | 0.476 | 5 |
| 381 | ROPN1L | 0.924 | 1.174814 | 0.848 | 0.915 | 0.138 | 5 |
| 382 | MLF1 | 0.917 | 0.961994 | 0.834 | 0.958 | 0.597 | 5 |
| 383 | APOBEC4 | 0.916 | 0.26573 | 0.832 | 0.806 | 0.008 | 5 |
| 384 | RSPH9 | 0.914 | 0.653467 | 0.828 | 0.879 | 0.389 | 5 |
| 385 | CCDC60 | 0.914 | 0.47341 | 0.828 | 0.861 | 0.156 | 5 |
| 386 | RP11-489D | 0.91 | 0.270886 | 0.82 | 0.824 | 0.063 | 5 |
| 387 | AGR3 | 0.909 | 1.141894 | 0.818 | 0.939 | 0.432 | 5 |
| 388 | FAM229B | 0.909 | 0.950069 | 0.818 | 0.952 | 0.789 | 5 |
| 389 | TCTEX1D4 | 0.905 | 1.248994 | 0.81 | 0.903 | 0.409 | 5 |
| 390 | ANXA1 | 0.903 | 1.167156 | 0.806 | 0.958 | 0.337 | 5 |
| 391 | EFCAB10 | 0.901 | 0.566865 | 0.802 | 0.873 | 0.262 | 5 |
| 392 | ZMYND10 | 0.9 | 0.9839 | 0.8 | 0.897 | 0.287 | 5 |
| 393 | ST6GALNA | 0.9 | 0.679849 | 0.8 | 0.952 | 0.315 | 5 |
| 394 | EFCAB1 | 0.896 | 1.142642 | 0.792 | 0.897 | 0.256 | 5 |
| 395 | C20orf201 | 0.896 | 0.320821 | 0.792 | 0.83 | 0.277 | 5 |
| 396 | RP11-1008 | 0.896 | 0.311004 | 0.792 | 0.794 | 0.063 | 5 |
| 397 | C1orf173 | 0.892 | 1.000332 | 0.784 | 0.867 | 0.2 | 5 |
| 398 | MAP1A | 0.892 | 0.572749 | 0.784 | 0.867 | 0.376 | 5 |

| 399 | DNAH2 | 0.891 | 0.307022 | 0.782 | 0.836 | 0.389 | 5 |
| --- | --- | --- | --- | --- | --- | --- | --- |
| 400 | LRRC9 | 0.89 | 0.32755 | 0.78 | 0.818 | 0.199 | 5 |
| 401 | TTC29 | 0.886 | 0.492417 | 0.772 | 0.921 | 0.552 | 5 |
| 402 | MGLL | 0.885 | 0.788739 | 0.77 | 0.988 | 0.5 | 5 |
| 403 | VWA3B | 0.884 | 0.361136 | 0.768 | 0.8 | 0.033 | 5 |
| 404 | RBP1 | 0.88 | 0.867333 | 0.76 | 0.903 | 0.69 | 5 |
| 405 | DNPH1 | 0.879 | 0.919539 | 0.758 | 1 | 0.92 | 5 |
| 406 | F5 | 0.879 | 0.48528 | 0.758 | 0.897 | 0.487 | 5 |
| 407 | CCDC78 | 0.878 | 1.006007 | 0.756 | 0.873 | 0.481 | 5 |
| 408 | PLTP | 0.877 | 1.102124 | 0.754 | 0.897 | 0.474 | 5 |
| 409 | SOD3 | 0.876 | 0.802707 | 0.752 | 0.982 | 0.627 | 5 |
| 410 | GJB7 | 0.875 | 0.269525 | 0.75 | 0.764 | 0.151 | 5 |
| 411 | ATP6V1G3 | 1 | 3.426549 | 1 | 1 | 0.149 | 6 |
| 412 | TMEM213 | 1 | 2.464042 | 1 | 1 | 0.299 | 6 |
| 413 | ATP6V0D2 | 1 | 2.02022 | 1 | 1 | 0.143 | 6 |
| 414 | FOXI1 | 1 | 2.018422 | 1 | 1 | 0.044 | 6 |
| 415 | DMRT2 | 1 | 1.588384 | 1 | 1 | 0.155 | 6 |
| 416 | HEPACAM2 | 1 | 1.261395 | 1 | 1 | 0.24 | 6 |
| 417 | MAP1B | 0.997 | 2.214633 | 0.994 | 1 | 0.436 | 6 |
| 418 | C12orf75 | 0.996 | 1.963581 | 0.992 | 1 | 0.338 | 6 |
| 419 | ATP6V1A | 0.995 | 1.386307 | 0.99 | 1 | 0.467 | 6 |
| 420 | LGALS3 | 0.993 | 2.239047 | 0.986 | 1 | 0.531 | 6 |
| 421 | KRT7 | 0.993 | 2.197181 | 0.986 | 1 | 0.585 | 6 |
| 422 | TFCP2L1 | 0.992 | 1.391445 | 0.984 | 1 | 0.467 | 6 |
| 423 | OXCT1 | 0.99 | 1.669367 | 0.98 | 1 | 0.475 | 6 |
| 424 | DEFB1 | 0.988 | 3.459269 | 0.976 | 1 | 0.54 | 6 |
| 425 | IL18 | 0.985 | 1.011029 | 0.97 | 1 | 0.255 | 6 |
| 426 | IDH2 | 0.983 | 1.259231 | 0.966 | 1 | 0.573 | 6 |
| 427 | PLCXD2 | 0.982 | 0.756003 | 0.964 | 0.974 | 0.279 | 6 |
| 428 | MAL | 0.979 | 1.445302 | 0.958 | 0.974 | 0.443 | 6 |
| 429 | IFI27L2 | 0.976 | 1.11086 | 0.952 | 1 | 0.551 | 6 |
| 430 | CLCNKB | 0.973 | 1.309534 | 0.946 | 0.974 | 0.167 | 6 |
| 431 | TSPAN7 | 0.969 | 0.77068 | 0.938 | 0.974 | 0.304 | 6 |
| 432 | TMSB4X | 0.968 | 1.316953 | 0.936 | 1 | 0.999 | 6 |
| 433 | RARRES2 | 0.966 | 1.563858 | 0.932 | 1 | 0.552 | 6 |
| 434 | C1orf168 | 0.966 | 1.03001 | 0.932 | 0.974 | 0.376 | 6 |
| 435 | S100A6 | 0.964 | 2.090997 | 0.928 | 1 | 0.59 | 6 |
| 436 | AC010982. | 0.964 | 1.404485 | 0.928 | 0.897 | 0.094 | 6 |
| 437 | THRSP | 0.961 | 0.698031 | 0.922 | 0.949 | 0.359 | 6 |
| 438 | IGF1 | 0.957 | 0.955816 | 0.914 | 0.949 | 0.383 | 6 |
| 439 | DGKI | 0.954 | 0.886191 | 0.908 | 0.923 | 0.285 | 6 |
| 440 | GPR110 | 0.949 | 0.642639 | 0.898 | 0.923 | 0.145 | 6 |
| 441 | CKB | 0.948 | 1.256414 | 0.896 | 1 | 0.541 | 6 |
| 442 | MT-CO2 | 0.948 | 1.206361 | 0.896 | 1 | 1 | 6 |
| 443 | CELF2 | 0.948 | 0.683439 | 0.896 | 0.923 | 0.149 | 6 |
| 444 | SMIM5 | 0.948 | 0.681762 | 0.896 | 0.949 | 0.321 | 6 |
| 445 | MUC1 | 0.947 | 0.976435 | 0.894 | 1 | 0.562 | 6 |
| 446 | SELM | 0.944 | 1.274351 | 0.888 | 0.974 | 0.554 | 6 |
| 447 | ATP6V0A4 | 0.944 | 0.983113 | 0.888 | 0.949 | 0.398 | 6 |
| 448 | ANGPTL1 | 0.944 | 0.367158 | 0.888 | 0.897 | 0.06 | 6 |

| 449 | FGF9 | 0.943 | 0.498797 | 0.886 | 0.897 | 0.165 | 6 |
| --- | --- | --- | --- | --- | --- | --- | --- |
| 450 | CHL1 | 0.942 | 0.414384 | 0.884 | 0.923 | 0.235 | 6 |
| 451 | NUPR1L | 0.941 | 0.975089 | 0.882 | 1 | 0.444 | 6 |
| 452 | FOXP1 | 0.94 | 0.833331 | 0.88 | 0.974 | 0.49 | 6 |
| 453 | PDE1C | 0.94 | 0.326549 | 0.88 | 0.872 | 0.071 | 6 |
| 454 | SMIM6 | 0.938 | 0.870552 | 0.876 | 1 | 0.479 | 6 |
| 455 | CA2 | 0.938 | 0.857538 | 0.876 | 0.974 | 0.706 | 6 |
| 456 | STAP1 | 0.937 | 1.543735 | 0.874 | 0.923 | 0.065 | 6 |
| 457 | GAS6 | 0.934 | 0.935103 | 0.868 | 0.949 | 0.452 | 6 |
| 458 | EFHD1 | 0.934 | 0.699047 | 0.868 | 0.897 | 0.254 | 6 |
| 459 | WDR72 | 0.933 | 0.64724 | 0.866 | 0.974 | 0.431 | 6 |
| 460 | PGM5-AS1 | 0.932 | 0.551548 | 0.864 | 0.897 | 0.295 | 6 |
| 461 | RBP1 | 0.931 | 1.046755 | 0.862 | 0.974 | 0.695 | 6 |
| 462 | CAMK2N1 | 0.931 | 1.02116 | 0.862 | 1 | 0.84 | 6 |
| 463 | ACAT1 | 0.931 | 0.960051 | 0.862 | 1 | 0.758 | 6 |
| 464 | ITIH5 | 0.929 | 0.902212 | 0.858 | 0.923 | 0.217 | 6 |
| 465 | S100A10 | 0.928 | 0.997058 | 0.856 | 1 | 0.955 | 6 |
| 466 | KCTD12 | 0.928 | 0.547207 | 0.856 | 0.974 | 0.38 | 6 |
| 467 | BLOC1S1 | 0.927 | 0.914437 | 0.854 | 0.974 | 0.695 | 6 |
| 468 | CHST2 | 0.922 | 0.406322 | 0.844 | 0.872 | 0.17 | 6 |
| 469 | SEPP1 | 0.918 | 1.208764 | 0.836 | 1 | 0.984 | 6 |
| 470 | TMEM61 | 0.917 | 0.584189 | 0.834 | 0.949 | 0.338 | 6 |
| 471 | PRSS23 | 0.917 | 0.43601 | 0.834 | 0.897 | 0.297 | 6 |
| 472 | CAV2 | 0.915 | 0.852757 | 0.83 | 0.923 | 0.293 | 6 |
| 473 | CA10 | 0.915 | 0.25714 | 0.83 | 0.846 | 0.176 | 6 |
| 474 | ITGA6 | 0.911 | 0.724328 | 0.822 | 0.949 | 0.471 | 6 |
| 475 | PHLDB2 | 0.911 | 0.627519 | 0.822 | 0.923 | 0.326 | 6 |
| 476 | PLLP | 0.91 | 0.894321 | 0.82 | 0.897 | 0.304 | 6 |
| 477 | TACC1 | 0.91 | 0.735119 | 0.82 | 0.974 | 0.532 | 6 |
| 478 | S100A2 | 0.91 | 0.664461 | 0.82 | 0.974 | 0.226 | 6 |
| 479 | ATP6V1B1 | 0.908 | 0.951826 | 0.816 | 0.897 | 0.181 | 6 |
| 480 | SCNN1A | 0.908 | 0.826149 | 0.816 | 0.974 | 0.599 | 6 |
| 481 | THBS1 | 0.905 | 0.698514 | 0.81 | 0.923 | 0.396 | 6 |
| 482 | CAV1 | 0.899 | 0.810464 | 0.798 | 0.949 | 0.362 | 6 |
| 483 | CNN3 | 0.898 | 0.901167 | 0.796 | 1 | 0.676 | 6 |
| 484 | TPRG1 | 0.894 | 0.601011 | 0.788 | 0.897 | 0.397 | 6 |
| 485 | SC5D | 0.892 | 0.669114 | 0.784 | 0.974 | 0.671 | 6 |
| 486 | OGDHL | 0.891 | 0.799304 | 0.782 | 0.897 | 0.262 | 6 |
| 487 | DHRS2 | 0.885 | 0.290112 | 0.77 | 0.821 | 0.293 | 6 |
| 488 | CFTR | 0.883 | 0.488595 | 0.766 | 0.923 | 0.36 | 6 |
| 489 | TIMP2 | 0.882 | 0.687838 | 0.764 | 0.872 | 0.318 | 6 |
| 490 | CA8 | 0.88 | 0.714038 | 0.76 | 0.949 | 0.679 | 6 |
| 491 | SLC25A3 | 0.878 | 0.857984 | 0.756 | 1 | 0.922 | 6 |
| 492 | CCL3L1 | 0.999 | 1.978918 | 0.998 | 1 | 0.032 | 7 |
| 493 | HLA-DRA | 0.998 | 2.96658 | 0.996 | 1 | 0.469 | 7 |
| 494 | HLA-DPA1 | 0.998 | 2.933327 | 0.996 | 1 | 0.277 | 7 |
| 495 | HLA-DPB1 | 0.997 | 2.787261 | 0.994 | 1 | 0.26 | 7 |
| 496 | CD74 | 0.994 | 2.475299 | 0.988 | 1 | 0.95 | 7 |
| 497 | HLA-DRB1 | 0.992 | 2.075187 | 0.984 | 1 | 0.506 | 7 |
| 498 | HCST | 0.989 | 1.167566 | 0.978 | 1 | 0.31 | 7 |

| 499 | IL1RN | 0.986 | 0.9407 | 0.972 | 1 | 0.15 | 7 |
| --- | --- | --- | --- | --- | --- | --- | --- |
| 500 | PDE4B | 0.981 | 0.964843 | 0.962 | 0.974 | 0.162 | 7 |
| 501 | TYROBP | 0.974 | 1.88904 | 0.948 | 0.974 | 0.151 | 7 |
| 502 | LST1 | 0.974 | 1.420798 | 0.948 | 0.974 | 0.249 | 7 |
| 503 | HLA-DQB1 | 0.973 | 1.519646 | 0.946 | 0.974 | 0.273 | 7 |
| 504 | BCL2A1 | 0.972 | 1.87071 | 0.944 | 0.974 | 0.167 | 7 |
| 505 | SRGN | 0.97 | 2.373261 | 0.94 | 0.974 | 0.258 | 7 |
| 506 | RGS10 | 0.964 | 1.367448 | 0.928 | 1 | 0.325 | 7 |
| 507 | LAPTM5 | 0.96 | 0.880593 | 0.92 | 0.949 | 0.205 | 7 |
| 508 | TMSB4X | 0.955 | 1.254353 | 0.91 | 1 | 0.999 | 7 |
| 509 | RNASE6 | 0.951 | 0.895871 | 0.902 | 1 | 0.404 | 7 |
| 510 | C15orf48 | 0.949 | 1.91707 | 0.898 | 0.949 | 0.196 | 7 |
| 511 | AIF1 | 0.948 | 1.682737 | 0.896 | 0.949 | 0.167 | 7 |
| 512 | FCER1G | 0.948 | 1.495001 | 0.896 | 0.949 | 0.252 | 7 |
| 513 | CD93 | 0.948 | 0.341926 | 0.896 | 0.949 | 0.241 | 7 |
| 514 | CCL3 | 0.947 | 3.85597 | 0.894 | 0.949 | 0.191 | 7 |
| 515 | IL1B | 0.947 | 3.046775 | 0.894 | 0.949 | 0.212 | 7 |
| 516 | GPR183 | 0.945 | 1.714909 | 0.89 | 0.949 | 0.173 | 7 |
| 517 | STC1 | 0.945 | 0.376068 | 0.89 | 0.974 | 0.523 | 7 |
| 518 | HLA-DQA1 | 0.944 | 1.500818 | 0.888 | 0.949 | 0.173 | 7 |
| 519 | TYMS | 0.942 | 0.439357 | 0.884 | 0.923 | 0.26 | 7 |
| 520 | ID2 | 0.938 | 1.736961 | 0.876 | 1 | 0.914 | 7 |
| 521 | PPP2R2C | 0.934 | 0.475697 | 0.868 | 1 | 0.451 | 7 |
| 522 | PPP1R1A | 0.933 | 0.402704 | 0.866 | 0.974 | 0.435 | 7 |
| 523 | THBD | 0.93 | 0.427988 | 0.86 | 0.923 | 0.283 | 7 |
| 524 | TOP2A | 0.928 | 0.269548 | 0.856 | 0.897 | 0.187 | 7 |
| 525 | CD22 | 0.926 | 0.512586 | 0.852 | 0.974 | 0.532 | 7 |
| 526 | RNASE1 | 0.925 | 0.374754 | 0.85 | 0.923 | 0.428 | 7 |
| 527 | HLA-DMA | 0.923 | 0.936593 | 0.846 | 0.949 | 0.364 | 7 |
| 528 | YBX1 | 0.923 | 0.752721 | 0.846 | 1 | 0.998 | 7 |
| 529 | SLC6A13 | 0.921 | 0.260942 | 0.842 | 0.974 | 0.528 | 7 |
| 530 | CCL4 | 0.919 | 3.308651 | 0.838 | 0.923 | 0.25 | 7 |
| 531 | TNFAIP2 | 0.918 | 0.759514 | 0.836 | 0.974 | 0.273 | 7 |
| 532 | DEGS2 | 0.918 | 0.501967 | 0.836 | 1 | 0.524 | 7 |
| 533 | F5 | 0.918 | 0.257592 | 0.836 | 0.974 | 0.496 | 7 |
| 534 | RAB31 | 0.914 | 0.92647 | 0.828 | 1 | 0.531 | 7 |
| 535 | FCER1A | 0.911 | 1.06179 | 0.822 | 0.897 | 0.05 | 7 |
| 536 | HAAO | 0.911 | 0.269498 | 0.822 | 0.949 | 0.425 | 7 |
| 537 | TNFAIP3 | 0.908 | 1.422165 | 0.816 | 1 | 0.496 | 7 |
| 538 | HAMP | 0.907 | 1.317262 | 0.814 | 0.897 | 0.192 | 7 |
| 539 | CD83 | 0.906 | 1.644895 | 0.812 | 0.923 | 0.429 | 7 |
| 540 | LHX1 | 0.906 | 0.316879 | 0.812 | 0.974 | 0.397 | 7 |
| 541 | PLAU | 0.904 | 0.732749 | 0.808 | 0.974 | 0.224 | 7 |
| 542 | NFKBIA | 0.903 | 1.490167 | 0.806 | 1 | 0.94 | 7 |
| 543 | C2orf88 | 0.903 | 0.346846 | 0.806 | 0.923 | 0.429 | 7 |
| 544 | ICAM1 | 0.902 | 0.981936 | 0.804 | 0.974 | 0.302 | 7 |
| 545 | AZGP1 | 0.901 | 0.596675 | 0.802 | 1 | 0.604 | 7 |
| 546 | PDLIM2 | 0.901 | 0.34114 | 0.802 | 0.974 | 0.532 | 7 |
| 547 | SERPING1 | 0.9 | 0.60931 | 0.8 | 1 | 0.699 | 7 |
| 548 | MS4A6A | 0.897 | 1.22384 | 0.794 | 0.897 | 0.207 | 7 |

| 549 | GATM | 0.896 | 0.639276 | 0.792 | 1 | 0.484 | 7 |
| --- | --- | --- | --- | --- | --- | --- | --- |
| 550 | GIPC2 | 0.895 | 0.368318 | 0.79 | 1 | 0.424 | 7 |
| 551 | GYPC | 0.894 | 0.695294 | 0.788 | 0.923 | 0.544 | 7 |
| 552 | ECM2 | 0.894 | 0.254937 | 0.788 | 0.974 | 0.36 | 7 |
| 553 | DPEP1 | 0.893 | 0.553901 | 0.786 | 1 | 0.58 | 7 |
| 554 | CCL2 | 0.892 | 0.806555 | 0.784 | 1 | 0.398 | 7 |
| 555 | KCNK15 | 0.892 | 0.421098 | 0.784 | 0.974 | 0.471 | 7 |
| 556 | SCGB1D4 | 0.89 | 1.011117 | 0.78 | 0.949 | 0.645 | 7 |
| 557 | SESN2 | 0.89 | 0.372482 | 0.78 | 0.974 | 0.316 | 7 |
| 558 | IL4R | 0.889 | 0.269858 | 0.778 | 0.923 | 0.257 | 7 |
| 559 | LGALS2 | 0.888 | 0.928658 | 0.776 | 1 | 0.671 | 7 |
| 560 | CPM | 0.888 | 0.727123 | 0.776 | 1 | 0.497 | 7 |
| 561 | PCK1 | 0.888 | 0.521486 | 0.776 | 0.974 | 0.524 | 7 |
| 562 | IGFBP5 | 0.886 | 0.499586 | 0.772 | 0.974 | 0.475 | 7 |
| 563 | NR2F2 | 0.885 | 0.567266 | 0.77 | 1 | 0.793 | 7 |
| 564 | IL1A | 0.885 | 0.390081 | 0.77 | 0.872 | 0.182 | 7 |
| 565 | FOSL1 | 0.884 | 0.341397 | 0.768 | 0.974 | 0.356 | 7 |
| 566 | SCARB1 | 0.884 | 0.27678 | 0.768 | 0.974 | 0.514 | 7 |
| 567 | NOSTRIN | 0.883 | 0.298673 | 0.766 | 0.974 | 0.473 | 7 |
| 568 | EPHA7 | 0.883 | 0.283192 | 0.766 | 0.974 | 0.597 | 7 |
| 569 | FTL | 0.881 | 1.39792 | 0.762 | 1 | 1 | 7 |
| 570 | CTTNBP2 | 0.881 | 0.289437 | 0.762 | 0.974 | 0.514 | 7 |
| 571 | FXYD5 | 0.88 | 0.965412 | 0.76 | 0.897 | 0.233 | 7 |
| 572 | SOX7 | 0.88 | 0.309959 | 0.76 | 0.949 | 0.38 | 7 |
| 573 | CXCL3 | 0.878 | 1.989007 | 0.756 | 0.949 | 0.361 | 7 |
| 574 | NSG1 | 0.877 | 0.530055 | 0.754 | 1 | 0.603 | 7 |
| 575 | EVI2B | 0.875 | 0.869304 | 0.75 | 0.872 | 0.009 | 7 |
